# Identification of Different Types of Spinal Afferent Nerve Endings That Encode Noxious and Innocuous Stimuli in the Large Intestine Using a Novel Anterograde Tracing Technique

**DOI:** 10.1371/journal.pone.0112466

**Published:** 2014-11-10

**Authors:** Nick J. Spencer, Melinda Kyloh, Michael Duffield

**Affiliations:** Discipline of Human Physiology and Centre for Neuroscience, School of Medicine, Flinders University of South Australia, Adelaide, Australia; University of California, Los Angeles, United States of America

## Abstract

In mammals, sensory stimuli in visceral organs, including those that underlie pain perception, are detected by spinal afferent neurons, whose cell bodies lie in dorsal root ganglia (DRG). One of the major challenges in visceral organs has been how to identify the different types of nerve endings of spinal afferents that transduce sensory stimuli into action potentials. The reason why spinal afferent nerve endings have been so challenging to identify is because no techniques have been available, until now, that can selectively label *only* spinal afferents, in high resolution. We have utilized an anterograde tracing technique, recently developed in our laboratory, which facilitates selective labeling of only spinal afferent axons and their nerve endings in visceral organs. Mice were anesthetized, lumbosacral DRGs surgically exposed, then injected with dextran-amine. Seven days post-surgery, the large intestine was removed. The characteristics of thirteen types of spinal afferent nerve endings were identified in detail. The greatest proportion of nerve endings was in submucosa (32%), circular muscle (25%) and myenteric ganglia (22%). Two morphologically distinct classes innervated myenteric ganglia. These were most commonly a novel class of intraganglionic varicose endings (IGVEs) and occasionally rectal intraganglionic laminar endings (rIGLEs). Three distinct classes of varicose nerve endings were found to innervate the submucosa and circular muscle, while one class innervated internodal strands, blood vessels, crypts of lieberkuhn, the mucosa and the longitudinal muscle. Distinct populations of sensory endings were CGRP-positive. We present the first complete characterization of the different types of spinal afferent nerve endings in a mammalian visceral organ. The findings reveal an unexpectedly complex array of different types of primary afferent endings that innervate specific layers of the large intestine. Some of the novel classes of nerve endings identified must underlie the transduction of noxious and/or innocuous stimuli from the large intestine.

## Introduction

Spinal afferent neurons play the major role in the detection and transmission of painful (noxious) and innocuous stimuli from visceral organs to the central nervous system (CNS) [1], [2], [3]. Whilst the origin of the cell bodies of spinal afferent neurons is well known to lie within dorsal root ganglia (DRG) [3], [4], [5], the sites of innervation of the nerve endings of spinal afferent neurons in different visceral organs have been considerably more difficult to identify. This is because the axons of spinal afferents always travel in mixed nerves as they innervate their target organ and run alongside all the other classes of extrinsic efferent (sympathetic and parasympathetic) and afferent (vagal) [6], [7], [8], [9], [10] or intestinofugal neurons [11]. This has meant that previous attempts to identify spinal afferent endings in the gut wall has been impeded by the fact that all the other classes of extrinsic nerve are also labeled [12]. Identifying spinal afferent nerve endings in the gastrointestinal (GI) tract has had an additional complication in that the GI-tract also has a complete nervous system of its own, known as the enteric nervous system [13], [14], [15]. Because some spinal afferent endings ramify within the enteric nervous system (ENS) [16], attempts to disentangle the nerve endings of spinal afferents from those of the ENS and extrinsic autonomic efferent or afferent nerve endings has proved exceptionally challenging. Another complication with identifying spinal afferent endings in the GI-tract, is that many of the neurochemical markers that are expressed in DRG (and hence are present in nerve endings within the gut wall) are also expressed in enteric neurons such as substance P [17], [18] and calcitonin gene related peptide (CGRP) [19]. Therefore, substance P or CGRP immunoreactivity alone cannot be used to identify spinal afferents in the gastrointestinal tract.

Previous in vitro studies have used anterograde tracing from mixed spinal nerve in vitro as they enter the gut wall [12], [20], [21], [22]. This approach has been used to successfully label a class of spinal afferent ending that lies on blood vessels in the small intestine [21], which function as high threshold mechanoreceptors, and low threshold rectal intraganglionic laminar endings (rIGLEs) in the rectum [16]. In the mouse colon, a class of intramuscular endings have also been identified in the smooth muscle [23] and myenteric ganglia [12]. However, in these studies, where nerve endings were anterogradely labeled from the rectal nerves, we could not be certain whether the labeled nerve endings were spinal afferents, since other classes of extrinsic nerves could also have been labeled, such as sympathetic efferents, parasympathetic efferents, intestinofugal afferents and also potentially vagal afferent endings. Functionally, it has been shown that deficits in low threshold, but not high threshold spinal afferents that innervate the mouse colorectum lead to substantially reduced nociception from this region [2], so understanding the characteristics of all the different types of spinal afferent endings in mouse colon can provide us with valuable new clues regarding nociception from this organ.

In this study, we have utilized a novel anterograde tracing technique, which involves injection of a neuronal tracer into small populations of DRGs in live mice. This approach allows, for the first time, the selective labeling of only spinal afferent endings in visceral organs [24]. We have used this approach to identify all the different types of spinal afferent endings that innervate the large intestine. The findings reveal an exceptionally complex range of distinct classes of spinal afferent endings within distinct anatomical layers of the gut wall.

## Materials and Methods

This study was carried out in strict accordance with the recommendations in the Guide for the Care and Use of Laboratory Animals of the National Health & Medical Research Council (NH&MRC) of Australia. The experimental protocol used in this study was approved by the Animal Welfare Committee of Flinders University of South Australia (ethics #784/11 & #861-13). All surgery was always performed under isoflurane anesthesia and every effort was made to minimize suffering. Male & female C57BL/6 mice (30–60 days old) were obtained from Laboratory Animal Services at the University of Adelaide, South Australia. Mice were anesthetized by isoflurane inhalation anesthetic (induced at 4%, maintained at 1.5% in oxygen). Whilst under anesthesia, a 1.5 cm long incision was made along the dorsal surface to expose the vertebral segments ∼2–3 cm rostral to the anus. An incision was made into the skeletal muscle lying superior to the dorsal root ganglia (DRG) and L6 & S1 DRG were exposed. A 5 µL Hamilton syringe (cat. #7634-01) Reno, Nevada, USA) was used to draw up 1 µL volume of biotinylated dextan biotin (10–20%, Cat #D1956, Molecular Probes, Eugene, Oregan, USA) that was made up in saline solution. This 1 µL volume solution was then injected into glass micropipettes (inner diameter: 1.5 mm; Cat #TW150-4; World Precision Instruments (WPI) that were pulled on a Flaming Brown micropipette puller (Model P-87; Sutter Instrument Co. USA) with tip diameters of approximately 5–10 microns. Microlectrodes were advanced independently into L6 and S1 DRG using a manual micromanipulator (Narashige model #M-4001002; Japan) then injected with 200–300 nL of this solution (containing dextran biotin 10–20% soln).

A custom made pressure ejection system (Biomedical Engineering, Flinders University) was used to apply highly controlled pulses of nitrogen (1s duration @ 0.3 Hz; 10–15psi) to glass microelectrodes for periods of 5–10 minutes. No physical damage was apparent when fine microelectrode tips were impaled into or retracted from DRGs in vivo. Once DRGs had been injected with dextran biotin, the skeletal muscle around the spine was then sutured with 2.0 suture (Dynek, Australia) and the wound site and incision closed with fine suture. The skin was closed using 7 mm wound clips (Cat #: 12032-07; Fine Science Tools, Canada) and animals allowed to recover for a minimum of 7 days post surgery, at which point they were euthanized by isoflurane inhalation overdose, followed by cervical dislocation (see ethics approval below). The entire colon was removed, incised along the longitudinal axis and pinned mucosal side uppermost, as a sheet preparation, in phosphate buffered saline (PBS). This full length sheet preparation (containing mucosa) was then fixed in 4% paraformaldehyde for 4–6 hours and then washed 3 times 10 minutes in dimethyl sulfoxide (DMSO). After this, the entire preparation was washed in PBS 3 times (each for 10 minutes duration) at which point the preparations were incubated (full thickness) for 2–3 hours in Cy3-conjugated Streptavidin (Cat #016 160 084, Jackson Immuno Research Laboratories Inc, West Grove, PA, USA). Preparations were then washed again 3 times (each time 10 minutes duration) in PBS and mounted in buffered glycerol.

After confirming biotin labeling in these specimens using a fluorescence microscope, slides were then unmounted washed again in PBS twice for each time for 20 minutes, then immersed in blocking solution for 1 hour (1% bovine serum albumin; BSA, 5% normal donkey serum & 1% triton X100) made up in PBS and incubated in primary antibody (CGRP Rabbit Peninsula, Cat. #A11729 at a concentration 1∶2000) for 48 hours. After primary incubation, the tissue was washed again 3 times (each time 10 minutes duration) in PBS and then incubated in secondary antibody (DaR CY5; Lot #105748; Jackson ImmunoResearch Laboratories, INC. West Grove, PA. U.S.A) at 1∶200 dilution for 2 hours. The entire full thickness colon was then washed again and mounted serosal side uppermost on slides that were cover slipped. Images were captured on a Nikon i50 fluorescence micrcoscope using Cy3 and Cy5 fluorescence filters. Image J software was used to measure projection lengths and diameters of fluorescently labeled neuronal structures.

### Limitations of anterograde tracing from DRG

Whilst there are many major advantages of the new anterograde tracing technique described in this study, there are some limitations. Firstly, the surgical technique is intricate and the procedure can be prone to excessive blood loss, if not properly controlled. Secondly, we found that at least 7 days was required to induce sufficient labeling in the viscera when using dextran biotin as an anterograde tracer. Periods less than 4 days post-DRG injection did not reveal strong labeling in any visceral organ. Thirdly, we are yet to identify any other neuronal tracers that induce clear labeling of fine nerve terminal morphology in any visceral organ similar to that induced with dextran biotin. Other tracers revealed axons, but not fine terminal morphology. We did test a variety of different tracers including injection of adeno-associated viruses (that express fluorescent proteins) and none revealed the fine morphological features of primary afferent nerve endings in any organ – even if extended periods of many weeks following DRG injections was allowed.

### Classification of different morphological types of spinal afferent nerve ending

To characterize the different types of nerve endings in the mouse large intestine, we used a classification scheme similar to that recently used to describe the different types of nerve endings in the corneal epithelium [25]. “Simple endings” have few branches with relatively long unbranched terminating axons. “Branching-type” endings consisted of numerous branching axons that lay parallel to each other and could project either circumferentially around the bowel, or in the longitudinal (rostro-caudal) axis. “Complex type” endings consisted of numerous varicose axons that arose from a single parent axon but the orientation of their terminal axons lacked any preferential directionality in the circumferential or longitudinal orientation. Axons of this class of ending projected in multiple directions following no apparent organized course (e.g. see Figure 2F in [25]). On rare occasions we also identified rIGLEs, which did not fit into the above classification scheme. Rectal IGLEs were identified by flattened, leaf-like endings which ramified within myenteric or submucosal ganglia, but lacked extensive varicosities along their axon terminals, as was common to the other morphological classes of endings described above.

### Confocal microscopy and analysis of data

Confocal imaging of full thickness specimens of mouse large intestine was performed on a Leica TCS SP5 laser scanning confocal microscope running Leica Application Suite Advanced Fluorescence Software (Leica Microsystems GmbH, Wetzlar, Germany) in sequential scanning mode. Confocal Z-stacks were taken with a 63× N.A. 1.4 oil immersion objective with a 2–3× digital zoom (pixel resolution 0.3 µm). The characteristics of nerve endings were analyzed using Image J software (version 1.45). In the Results section, the use of “N” in the results sections refers to the number of animals on which observations were made.

## Results

In total, 45 mice were anesthetized and L6 and S1 DRGs unilaterally injected with dextran biotin (see Methods). Seven days post-surgery, the entire colon was fresh-fixed with mucosa intact (see Methods) and processed for CGRP immunohistochemistry. Because we strategically injected minute quantities of tracer into DRGs, this had the major benefit of labeling very few axons entering the large intestine. This allowed us to visualize the projection of a single primary afferent axon as it entered the large intestine and then follow their precise trajectory throughout the full thickness layers of the large bowel. It was determined that all axons entered the large bowel within the terminal 30 mm from the anus after L6 and S1 DRG injections. We found that once a single labeled axon entered the bowel it typically weaved through many rows of myenteric ganglia considerable distances (many mm) in a rostro-caudal direction, consistent with the findings in our recent technical report see, [24]. Injection of saline alone (without the dextran biotin) into DRGs never revealed any labeling in the large intestine.

In full thickness specimens of freshly-fixed whole colon (containing mucosa) it was possible to determine all the major sites of innervation of primary afferent nerve endings. These were found to be the submucosa (32%), myenteric ganglia 26% (which included a novel class of ending in internodal strands –4%) and the circular muscle (25%). Within the CM and submucosa, there were three consistent but morphologically distinct types of spinal endings (see further below). No nerve endings were ever identified in the serosa.

### Intraganglionic varicose endings (IGVEs) in myenteric ganglia

**Figure 1:**
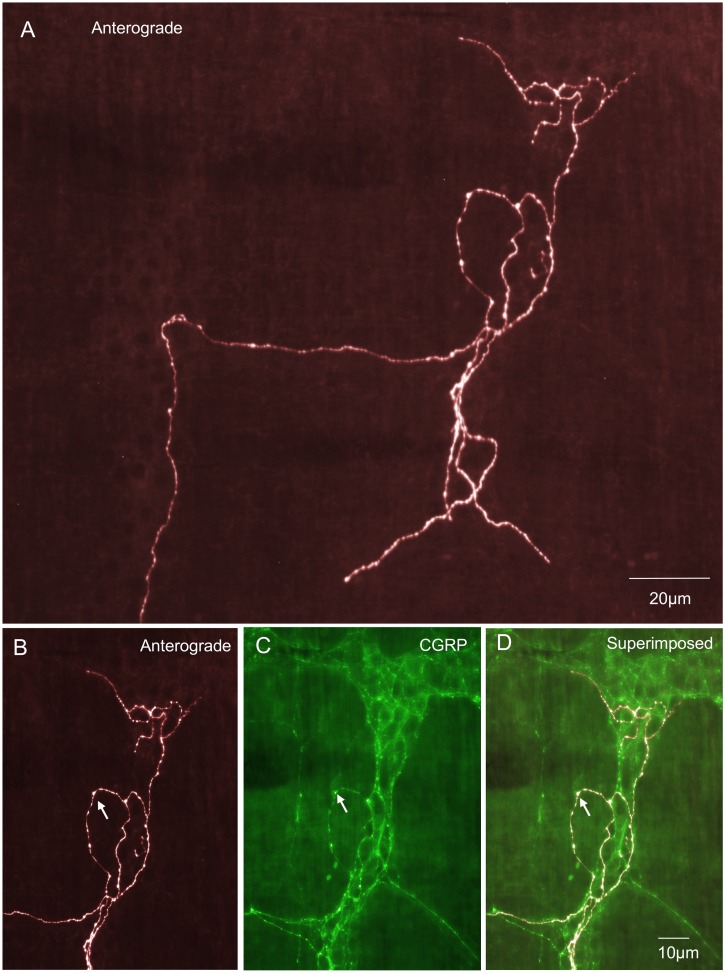
Anterograde labeling from lumbosacral DRG in vivo reveals intraganglionic varicose endings (IGVEs) in a myenteric ganglion. A, shows a single axon of an anterogradely labeled spinal afferent that enters a myenteric ganglion then bifurcates into multiple other axon terminals each of which ramifies extensively throughout the ganglion, but lacks any complex nerve terminal specialization. The single axon that enters the ganglion subdivides to form 6 different endings that all terminate at different sites within the same ganglion. B, shows the upper segment of this myenteric ganglion and the spinal afferent that ramifies throughout this region. The arrow indicates a single axon that exits the ganglion briefly before returning back into the same ganglion. C, shows CGRP immunoreactivity of the region shown in panel B. The arrow in C indicates the axon in panel B (indicated by the arrow) is CGRP immunoreactive. Panel D, shows a superimposed image of panels B & C. This IGVE is CGRP immunoreactive (compare arrows in panels B, C & D).

**Figure 2:**
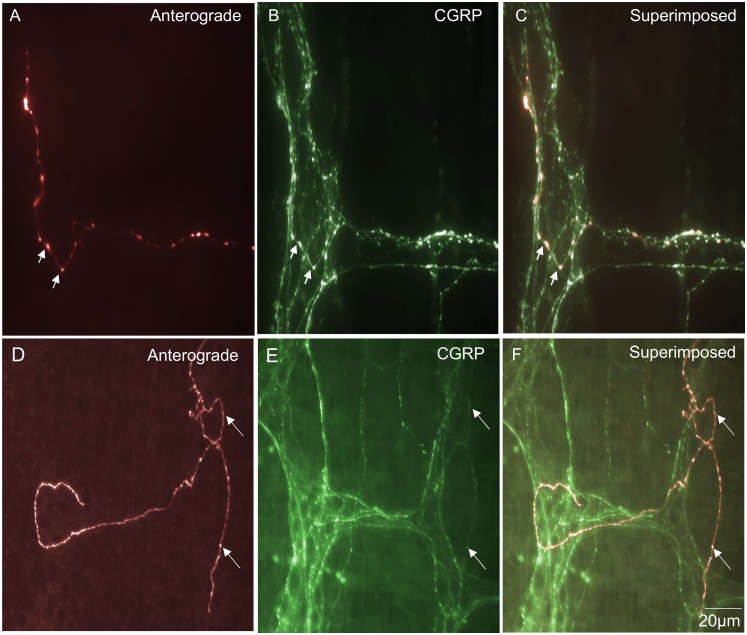
Anterograde labeling from lumbosacral DRG in vivo reveals two different examples (from two different animals) of single IGVEs that lack extensive bifurcations throughout the myenteric ganglion. A, shows an anterogradely labeled spinal afferent IGVE that consists of a single varicose axon without complex axonal ramifications throughout the ganglion. The two arrows indicate two discrete varicosities along its axon. B, shows CGRP immunoreactivity of the image shown in A. The two varicosities indicated by the arrows in panel B can be seen to be immunoreactive to CGRP which are the same varicosities as indicated by the arrows in panel A. C, shows a superimposed image of A and B. The arrows indicate colocalization of CGRP in the anterogradely labeled varicosities. Panel D, shows a single IGVE from a different animal with few varicosities along its axon terminal and lacking extensive bifurcations throughout the ganglion. The two arrows indicate an axon that has exited the ganglion briefly before returning back into the same ganglion. E, shows CGRP immunoreactivity of the myenteric ganglion shown in panel D and that the anterogradely labeled axon indicated by the arrows in D is CGRP positive. Panel F, shows a superimposed image of D & E. The arrows show colocalization of the anterogradely labeled IGVE and CGRP immunoreactivity.

**Figure 3:**
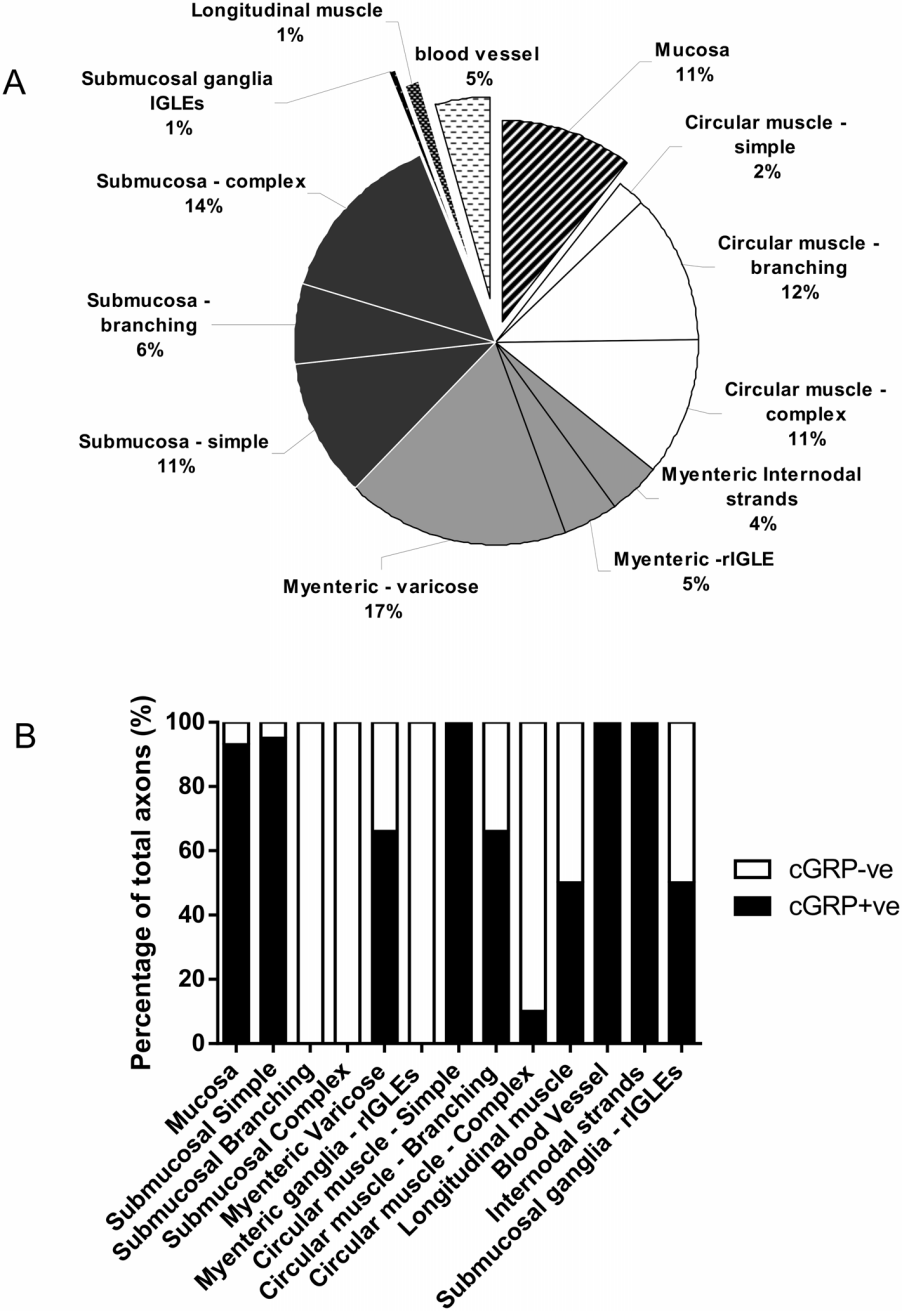
Graphical representation of the relative proportion of spinal afferent nerve endings that innervate distinct anatomical sites within the large intestine. A, shows that the majority of nerve endings were identified in the circular muscle layer (3 distinct types present), submucosa (3 distinct types present) and myenteric ganglia including internodal strands (3 distinct types). Very few endings were identified in submucosal ganglia and longitudinal muscle. 11% of all anterogradely labeled spinal afferent endings innervated the mucosa. B, shows the percentage of labeled spinal afferent endings that were immunoreactive (positive and negative) for CGRP. Some classes of spinal afferent nerve endings such as simple endings in the CM layer, those in internodal strands and blood vessels were always CGRP immunoreactive. However, rIGLEs in myenteric ganglia, complex-type endings in submucosa and branching-type endings in submucosa were never found to be CGRP positive.

The most common type of primary afferent nerve ending in myenteric ganglia was identified as an intraganglionic varicose ending (IGVEs) (Fig. 1A). These have not been identified previously probably because of their similarity to sympathetic efferent nerve endings and the lack of techniques available to selectively label this type of sensory fibre. In total, 39 IGVEs were identified (N = 14) that were found to arise from a single spinal afferent axon. In a single myenteric ganglion, IGVEs were found to ramify a mean distance of 122±19 µm in the circumferential axis and 124±17 µm in the rostro-caudal axis; 39 endings, N = 14. Another major feature of IGVEs was that a single spinal afferent axon that entered a myenteric ganglion often split and gave rise to multiple axon terminal arbors. This can be seen in Figure 1A where a single axon enters a myenteric ganglion, but this axon subdivides into at least 5 different terminal endings, all within the same ganglion. Most IGVEs consisted of varicosities along their axons, but lacked any complex specializations at their ending (Fig. 1 & 2). Some IGVEs consisted of relatively bare axons that lacked numerous large varicosities and did not have extensive branches or ramifications throughout the ganglion (Fig. 2D). Another feature of many IGVEs was that these axons often projected out of a single ganglion (and into the extraganglionic space), but then within a short distance, projected back into the same ganglion (see Fig. 1B–D & 2D). In total, IGVEs comprised 22% of all the spinal afferent endings identified in the mouse colon. The majority (66%) of all IGVEs were found to be CGRP positive (Fig. 3B). The diameter of the axon terminal of IGVEs was approximately 1 µm (Table 1).

**Table 1:** Characteristics of different classes of spinal afferent nerve endings identified in mouse large intestine following anterograde labeling from DRG in vivo.

Type of ending	Axon Diam(µm)	VaricosityDiam (µm)	VaricosityLength(µm)	Physical ExtentCircumferential(µm)	Physical extentcaudal-rostral(µm)
LM	1±0.5(n = 2; N = 2)	2±1(n = 2; N = 2)	3±1(n = 2; N = 2)	234±23(n = 2; N = 2)	546±18(n = 2; N = 2)
MyentericVaricose	1±0.5(n = 31; N = 14)	2±1(n = 31; N = 14)	2±1(n = 39; N = 14)	122±19(n = 39; N = 14)	124±17(n = 39; N = 14)
MyentericrIGLE	1±0.5(n = 10; N = 5)	3±1(n = 10; N = 5)	4±1(n = 10; N = 5)	91±16(n = 10; N = 5)	62±14(n = 10; N = 5)
InternodalStrand	1±0.5(n = 9; N = 3)	2±1(n = 9; N = 3)	4±1(n = 9; N = 3)	ND	ND
CM-Complex	1±0.5(n = 17; N = 11)	2±1(n = 17; N = 11)	4±1(n = 17; N = 11)	439±77(n = 17; N = 11)	250±65(n = 17; N = 11)
CM-Simple	1±0.5(n = 5; N = 3)	2±1(n = 5; N = 3)	3±1(n = 5; N = 3)	151±70(n = 5; N = 3)	19±16(n = 5; N = 3)
CM-Branching	1±0.5(n = 26; N = 10)	2±1(n = 26; N = 10)	4±1(n = 26; N = 10)	759±126(n = 26; N = 10)	176±18(n = 26; N = 10)
Submucosal-Complex	1±0.5(n = 31; N = 8)	2±1(n = 31; N = 8)	4±1(n = 31; N = 8)	264±23(n = 31; N = 8)	380±29(n = 31; N = 8)
Submucosal-Simple	1±0.5(n = 24; N = 9)	2±1(n = 24; N = 9)	3±1(n = 24; N = 9)	256±48(n = 24; N = 9)	281±64(n = 24; N = 9)
Submucosal-Branching	1±0.5(n = 14; N = 7)	2±1(n = 14; N = 7)	4±1(n = 14; N = 7)	177±39(n = 14; N = 7)	743±49(n = 14; N = 7)
SubmucosalGanglion rIGLE	1±0.5(n = 2; N = 2)	2±1(n = 2; N = 2)	6±1(n = 2; N = 2)	ND	ND
Bloodvessels	1±0.5(n = 10; N = 4)	2±1(n = 10; N = 4)	3±1(n = 10; N = 4)	243±80(n = 10; N = 4)	95±50(n = 10; N = 4)
Mucosa	1±0.5(n = 23; N = 7)	2±1(n = 23; N = 7)	3±1(n = 23; N = 7)	228±36(n = 23; N = 7)	325±55(n = 23; N = 7)

### Rectal intraganglionic laminar endings (rIGLEs) in myenteric ganglia

On relatively rare occasions, rIGLEs were identified in myenteric ganglia located within the terminal 15 mm of colorectum. They occupied only 4% of all the types of endings identified. To the best of our knowledge rIGLEs have not been identified previously in mouse large intestine. In total, 10 rIGLEs were identified in 5 out of 45 mice studied. The morphology of rIGLEs that ramified within a single myenteric ganglion consisted of flattened laminar endings and were never immunoreactive to CGRP, similar to IGLEs described in guinea-pigs [16]. All identified rIGLEs were located within the most distal region of colorectum that was directly innervated by rectal nerves. Although there is no anatomically defined rectal chamber in mice, we define this region as the terminal segment of large intestine that is innervated by the rectal nerves. This encompasses a region approximately 20 mm from the anal sphincter. The diameter of the single axon that gives rises to an rIGLE measured just prior to the formation of laminar dendrites in a myenteric ganglion was approximately 1 µm (Table 1).

### Spinal afferent endings in internodal strands

**Figure 4:**
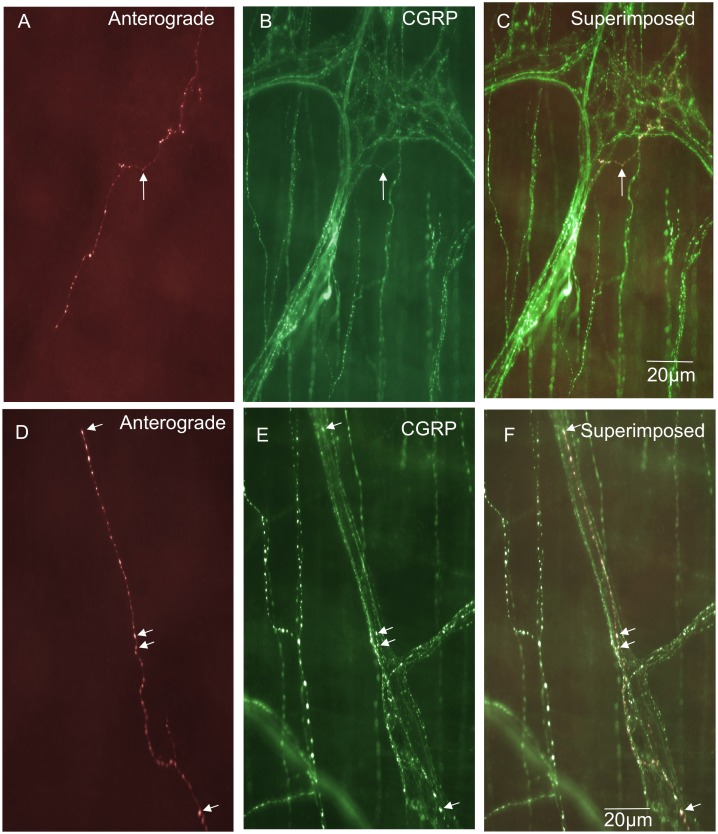
Anterograde labeling from lumbosacral DRG in vivo reveals spinal afferent nerve endings identified in internodal strands that run between myenteric ganglia. A, shows an anterogradely labeled single axon and varicose nerve ending terminating in an internodal strand. B, shows the CGRP image of the panel shown in A. This anterogradely labeled ending is CGRP positive. The arrow in panel B indicates the same spinal afferent axon as in panel A. This CGRP positive axon exits the myenteric ganglion briefly before returning back into the internodal strand. The arrow in panel C, shows a superimposed image of panels A & B. The arrow in panel C indicates a superimposed image of the CGRP positive axon in panel B and the anterogradely labeled axon in panel A. Panel D, shows a second example from a different animal of a single spinal afferent axon that terminates in an internodal strand. E, shows CGRP immunoreactivity of the same region in panel A. The three arrows in panel A indicate three discrete varicosities which are also CGRP positive (see arrows) in panel E. F, shows a superimposed image of panels D & E. The arrows in panel F indicate in this superimposed image of anterogradely labeled varicosities and CGRP immunoreactivity.

In 4% of all nerve endings identified, a unique population of endings was identified in internodal strands that ran between myenteric ganglia (n = 9; N = 3; see Fig. 4). These types of endings have not been identified previously, but consistently arose from a single axon with multiple varicosities along their axon terminal (Fig. 4). No axon terminal arbors were identified that arose from this type of ending. That is, they lacked any complex specialization or branching at their endings (Fig. 4). Varicosities located at nerve ending of this class of afferent were not morphologically distinct from varicosities located along the length of the axon. All spinal endings identified in internodal strands were all immunoreactive to CGRP (Fig. 3B). The diameter of the axon at the nerve terminal of this class of spinal afferent was approximately 1 µm (Table 1).

### “Complex-type” spinal afferent endings in circular muscle

**Figure 5:**
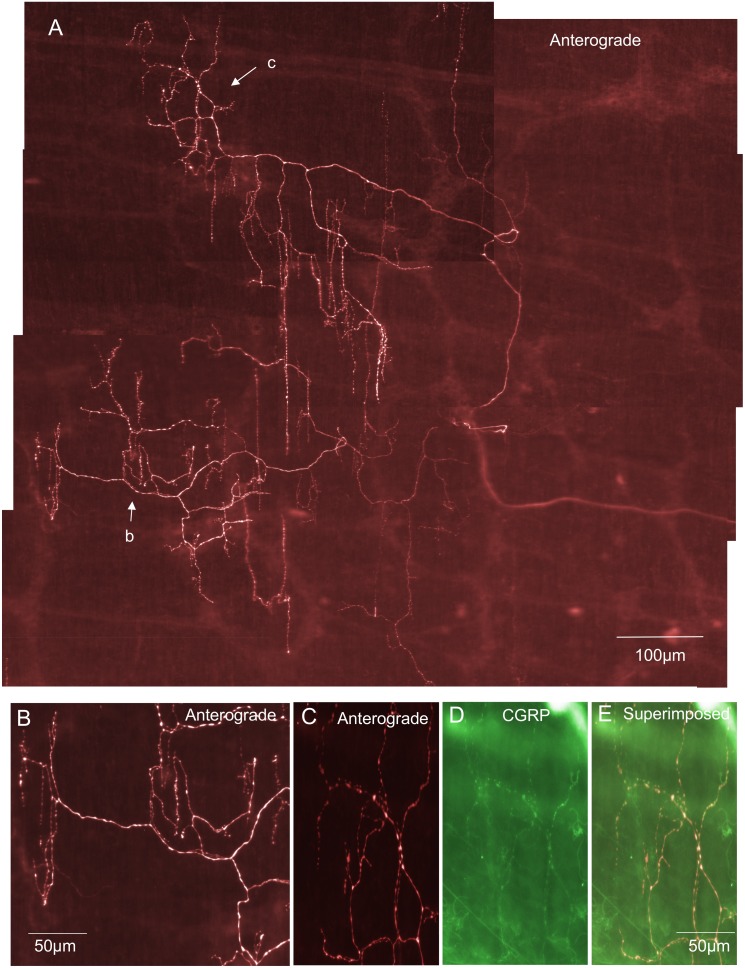
Anterograde labeling from lumbosacral DRG in vivo reveals a “complex-type” spinal afferent nerve ending in the circular muscle layer. A, shows a montage of a single spinal afferent axon that traverses through many myenteric ganglia, then projects into the CM layer forming a highly complex axonal arbor with multiple varicose branch points. These axon terminals do not align in any preferential orientation with the CM layer. Panel B, shows an expanded portion of the region indicated by the arrow b in panel A. Arrow C in panel A indicates part of the region shown in panel C on expanded scale. Panel D, shows the CGRP immunoreactivity of the region shown in panel C. Panel E, shows a superimposed image of panels C & D. This complex type ending is CGRP positive.

Three distinct morphological classes of spinal afferent ending were identified in the circular muscle, comprising 25% of all the spinal afferent endings in mouse large intestine. Of these, 11% were found to have “complex-type” morphology, 12% were found to have “branching-type” morphology and 2% were identified as “simple-type” endings. One of the major classes in the CM layer was the complex-type of ending, which arose from a single axon that projected out of a myenteric ganglion, then into the CM layer branching extensively, forming multiple axonal arbors within the muscle (Fig. 5A). These complex-type endings ramified a mean distance of 439±77 µm in the circumferential axis and 250±65 µm in the rostral-caudal axis (Table 1). Complex-type endings comprised a total of 11% of all the endings identified (Fig. 3A) but only 10% were CGRP positive (Fig. 3B). A unique defining feature of complex-type endings was that their axons branched extensively within the circular muscle, but with no preferential orientation to the muscle fibres (Fig. 5A). This meant that a single axon could split and subdivide, forming multiple branching varicose axons that projected circumferential, longitudinal and orthogonal to the circular muscle fibres (see Fig. 5A). To confirm complex-type endings terminated in the CM layer, we sharp dissected strands of CM away from the myenteric ganglia and this process always removed all these endings. The diameter of the axon terminal of this class of spinal afferent prior to branching extensively in the CM was approximately 1 µm (Table 1).

### “Simple type” endings in the circular muscle layer

**Figure 6:**
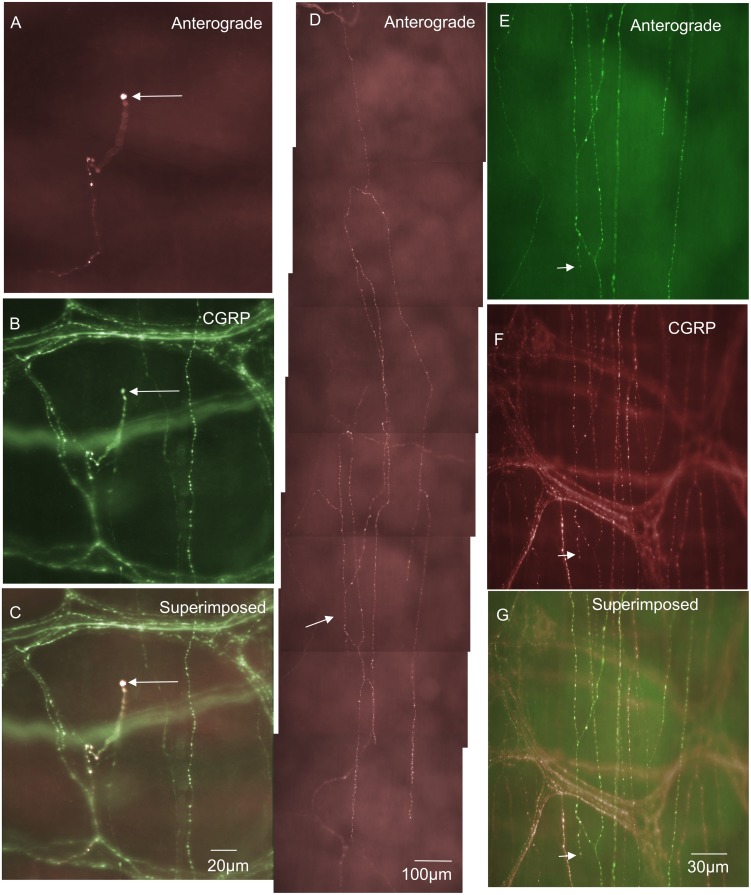
Anterograde labeling from lumbosacral DRG in vivo reveals two different types of spinal afferent nerve endings that innervate the circular muscle layer. A, shows a “simple class” of ending that consists of a single axon that does not branch, but rather projects out of a myenteric ganglion and then a short distance into the CM, terminating in a varicose ending (see arrow). B, shows CGRP immunoreactivity of the image shown in panel A. The arrow in panel B indicates the same varicose ending as shown by the arrow in panel A. This nerve ending is CGRP positive. Panel C, shows a superimposed image of A & B with the arrow indicating the same varicosity as in panels A & B. Panel D shows a “branching-type” ending in the CM layer. This ending arose from a single parent axon, that subdivided into multiple other varicose axons running parallel to the CM fibres. The region indicated by the arrow is shown in expanded scale in panel E. The arrow in panel E indicates a varicose ending in the CM layer. Panel F, shows a CGRP image of the region shown in panel E. The arrow in panel F indicates that the same ending as shown in panel E (see arrow) which is CGRP positive. Panel G, shows a superimposed overlay of panels E & F. All the anterogradely labeled axons can be seen to be CGRP positive. There are other CGRP immunoreactive axons that were not identified by anterograde labeling.

These endings consisted of single axons that were varicose in nature and innervated the CM parallel to the CM fibres (Fig. 6). Simple type endings could be seen emanating from internodal strands and projecting into the CM layer, then terminating as simple varicose endings. They had a mean projection length of 151±70 µm around the circumferential axis of the colon and mean projection distance of 19±16 µm in the rostro-caudal axis. This type of primary afferent ending comprised a total of 2% of all the nerve endings identified (Fig. 3A) and all simple endings were found to be immunoreactive to CGRP (Fig. 3B & Fig. 6B & Fig. 6C). The diameter of the axon at the nerve terminal of this class of spinal afferent was approximately 1 µm (Table 1).

### “Branching type” endings in the circular muscle layer

Branching type endings innervated the CM either as a single axon that emanated from a myenteric ganglion or internodal strand, which then bifurcated and branched into multiple neighbouring axons that always lay parallel to the CM fibres (Fig. 6D). In total, 12% of all spinal afferent endings identified were branching type endings (Fig. 3A) and all were CGRP positive (Fig. 6E & 6F). This type of primary afferent ending in CM had mean projection lengths around the circumferential axis of 759±126 µm and 176±18 µm in the rostro-caudal axis (see Table 1). The diameter of this class of nerve axon in the CM was approximately 1 µm (Table 1).

### Longitudinal muscle

**Figure 7:**
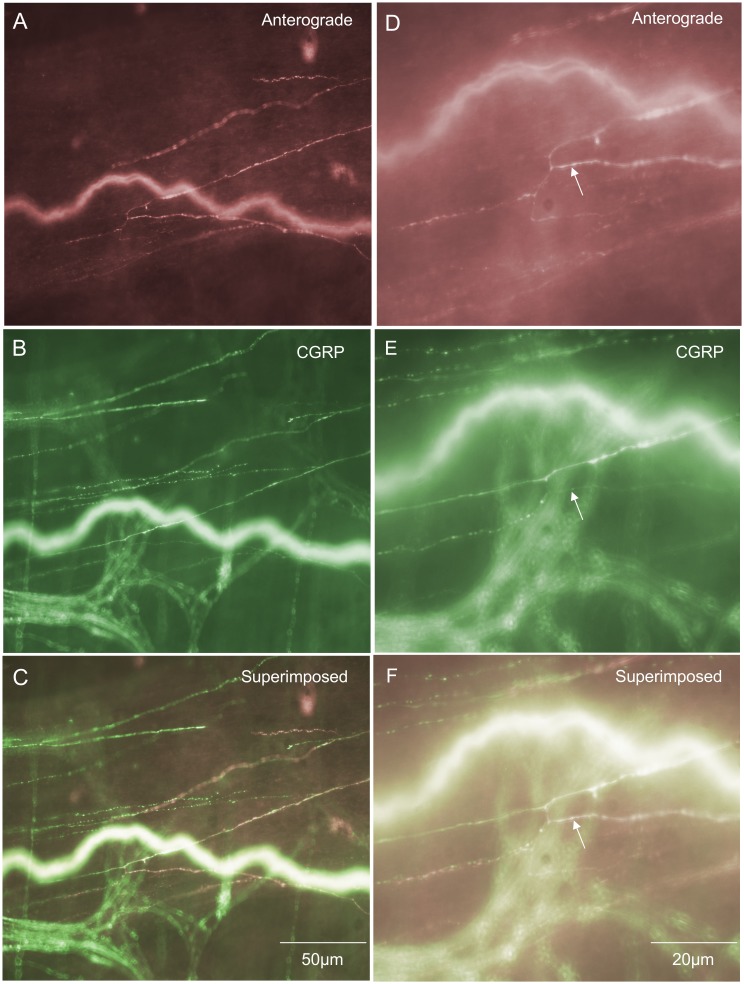
Anterograde labeling from lumbosacral DRG in vivo reveals on rare occasions spinal afferent nerve endings and axons in longitudinal muscle. A, shows branching varicose axons that terminate parallel to the LM fibres. B, shows CGRP immunoreactivity of the same region shown in A. Panel C, superimposed image of panels A & B. Panel D, shows a higher magnification image of part of the anterogradely labeled fibres shown in C. The arrow indicates part of the axon terminal lying in the LM layer. E, shows CGRP immunoreactivity of the same region shown in panel D. The arrow indicates CGRP immunoreactivity of the same anterogradely labeled endings in panel D. Panel F shows a superimposed image of panels D & E. The anterogradely labeled axons in D were immunoreactive to CGRP (see arrow).

Spinal afferent endings in the longitudinal muscle layer were extremely rare. This type of ending comprised only 1% of all the nerve endings identified (from N = 45 mice). In the small number of endings identified in the LM, it was found these endings branched within the muscle layer and a proportion were immunoreactive to CGRP (Fig. 3 & Fig. 7). The diameter of this class of nerve axon, close to its ending in the LM layer was approximately 1 µm (Table 1).

### Blood vessel spinal afferent endings

**Figure 8:**
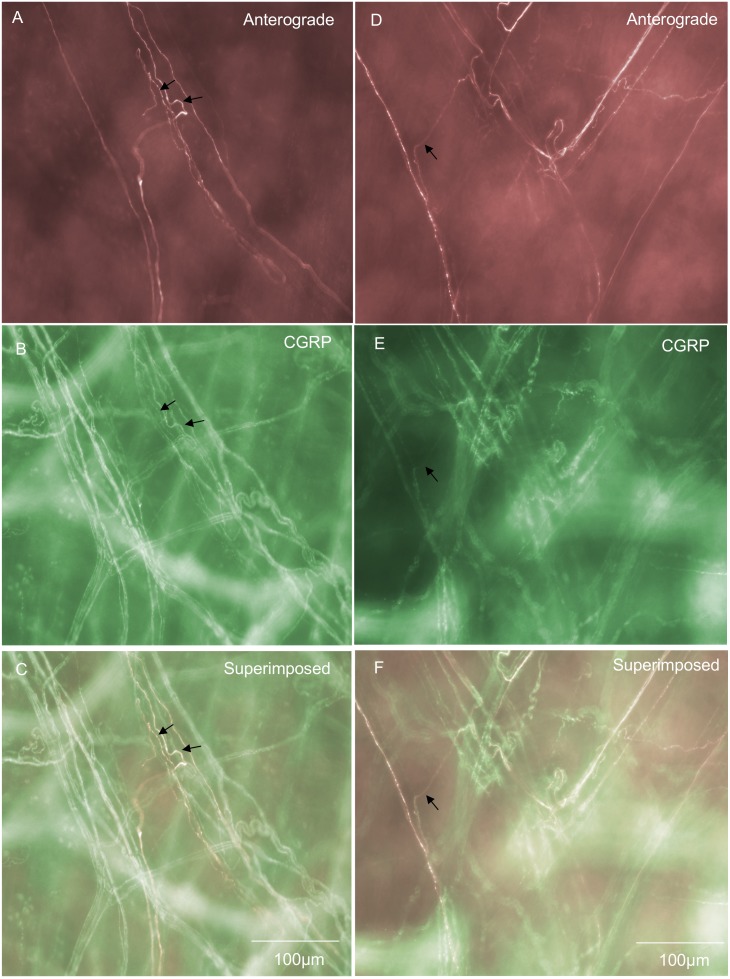
Anterograde labeling from lumbosacral DRG in vivo reveals spinal afferent endings and axons in blood vessels. A, shows a single axon and fine nerve ending that terminates without any complex morphology and few varicosities along its axon. The arrows in A indicate the trajectory of single anterogradely labeled axons. B, shows CGRP immunoreactivity of the same region shown in panel A. The arrows in B show positive CGRP immunoreactivity of the anterogradely labeled axons in panel A (indicated by arrows). Many other CGRP immunoreactive nerve fibres project along this blood vessel but were not identified after anterograde labeling. Panel C, shows a superimposed image of panels B & C, with the arrows indicating colocalization between anterogradely labeled axons and CGRP. Panel D, shows a blood vessel from a different animal that bifurcates and branches into two other vessels. Spinal afferent axons ramify along and around these blood vessels. A single axon (see arrow) can be seen ramifying over one of the blood vessels. E, shows CGRP immunoreactivity of the same region shown in panel D. The arrow indicates the same axon as in panel D (see arrow) and this axon is CGRP positive. F, shows a superimposed image of panels D & E and the arrow shows colocalization of the anterogradely labeled axons with CGRP.

Nerve endings on blood vessels comprised 5% of all spinal afferent endings in the large intestine (Fig. 8C & 8D). This type of ending consisted of fine axons with few varicosities that ramified over the blood vessels and had tapered endings that lacked any complex morphological structure (Fig. 8A). The diameter of this class of nerve axon, close to the ending in the vessel was approximately 1 µm (Table 1).

### “Branching-type” endings in submucosa

**Figure 9:**
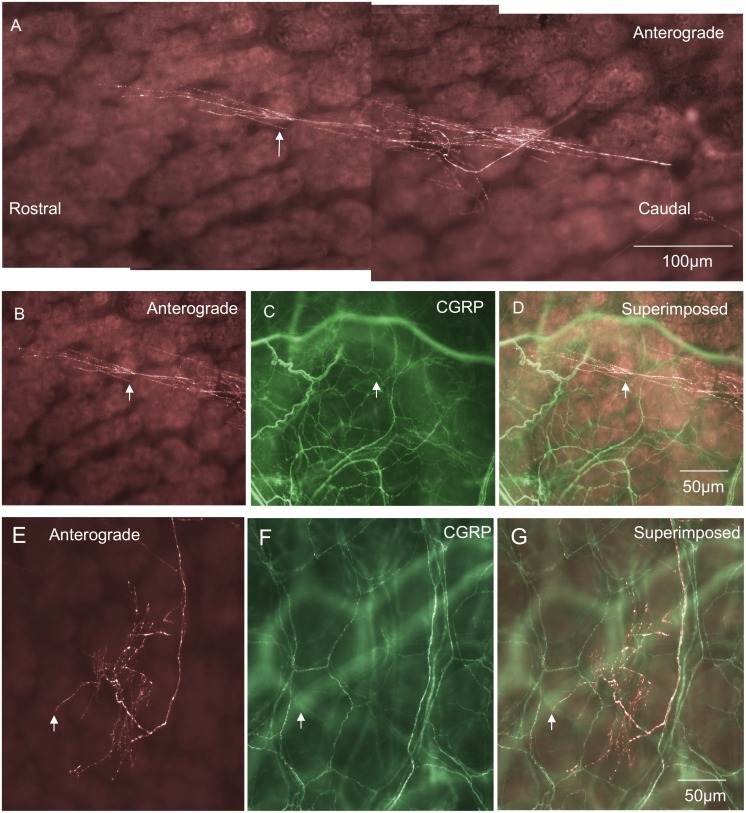
Anterograde labeling from lumbosacral DRG in vivo reveals two distinct types of spinal afferent nerve ending identified in the submucosa. A, shows a “branching-type” spinal afferent nerve ending in the submucosa. This ending ramifies extensively with many branching varicose axonal processes that align preferentially in the rostral-caudal axis, within the submucosa. The region indicated by the arrow in panel A is shown on expanded scale in panel B. The nerve endings indicated by the arrow in panel B are not CGRP positive (compare with arrow in panel C). Panel C shows CGRP immunoreactivity of the region shown in panel B. The arrow shows a lack of CGRP immunoreactivity of the ending indicated by the arrow in panel B. Panel D, shows a superimposed image of panels B & C. Panel E, shows a “complex-type” spinal afferent ending in the submucosa at the same level as the base of the Crypts of Lieberkhun. This complex type ending arises from a single spinal afferent axon and then ramifies into a complex structure with multiple varicose axons that branch in no apparent preferential orientation. The arrow in panel E indicates a varicose axonal ending that is not CGRP positive (compare with arrow at the same region in panel F). F, shows CGRP immunoreactivity of the same region shown in panel D. The arrow in panel F shows a lack of CGRP immunoreactivity of the ending in panel E. G, shows a superimposed image of E & F. It is clear that this complex-type spinal afferent ending is not CGRP immunoreactive - compare arrows in panels E, F & G.

Three distinct morphological classes of spinal afferent were found to exist in the submucosa. Collectively, these three types of endings comprised 32% of all spinal afferent endings in the large bowel. One of these classes was identified as a “branching-type” ending which comprised 6% of all spinal afferent endings. This class of ending arose from a single axon, then branched into numerous varicose axons that projected along the rostro-caudal axis (Fig. 9A). Branching-type endings ramified a mean of 743±49 µm in the rosto-caudal axis and 177±39 µm in the circumferential axis (Table 1). Interestingly, none of these branching-type endings were CGRP immunoreactive (from 14 endings; N = 7) (See Fig. 9C & 9D; Fig. 3B). The diameter of the axon of this class of spinal afferent, measured immediately prior to the formation of multiple branching axons in the rostro-caudal axis was approximately 1 µm (Table 1).

### “Complex-type” endings in submucosa

The second major type of afferent ending identified in the submucosa was classified as “complex-type” (Fig. 9E). These endings comprised 14% of all the nerve endings identified and were never found to be CGRP positive (Fig. 9F & 9G). These endings arose from a single axon at the level of the base of the Crypts of Lieberkhun, but subdivided into multiple branching axons that projected in no preferential direction (see Fig. 9E). Complex type endings projected a mean distance of 380±29 along the rostral-caudal axis and 264±23 in the circumferential axis (n = 31 endings; N = 9 animals). The diameter of the axon of this class of nerve ending was approximately 1 µm (see Table 1).

### “Simple type” endings in submucosa

**Figure 10:**
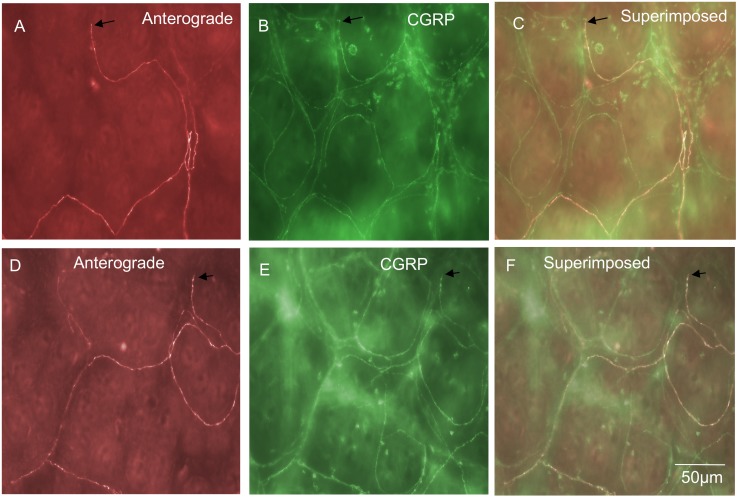
Anterograde labeling from lumbosacral DRG in vivo reveals “simple type” spinal afferent nerve endings that innervate the submucosa at the level of the Crypts of Lieberkhun. A, a single spinal afferent axon and its nerve ending is shown that encircles the base of the Crypts then terminates as a simple ending which lacks complex morphology. The arrow indicates the simple ending of this spinal afferent. B, shows CGRP immunoreactivity of the same region as shown in panel A. The arrow in panel B shows the presence of CGRP immunoreactivity in the same ending indicated by the arrow in panel A. Panel C, shows a superimposed image of panels A & B. The arrow indicates colocalization of CGRP in this primary afferent ending. B, it is noteworthy that extensive CGRP immunoreactive axons encase the Crypts and these axons have consistently few or no varicosities along their axons. D, shows a single spinal afferent axon from another animal and its ending that terminates around a single Crypt. The arrow indicates the simple nerve ending. E, shows CGRP immunoreactivity of the region shown in D. The arrow indicates CGRP immunoreactivity of the same ending as in panel D. Panel F, shows a superimposed image of D & E. The ending in D is CGRP positive (see arrow).

The third major class of ending identified in the submucosa was defined as “simple-type” ending that comprised 11% of all spinal afferent endings in the large intestine. This class of ending consisted of single axons that were found to encircle the base of the Crypts of Leiberkhun (Fig. 10A & 10D). This type of ending was readily identified because these simple-type endings developed a circular type pattern as their axons weaved for up to 256±48 µm in the circumferential axis and 281±64 µm in the rostral-caudal axis, wrapping around multiple crypts over this projection distance. A unique consistent feature of simple-type endings was that they were consistently non-varicose, or very weakly varicose in nature (Fig. 10); and did not terminate with any specialized morphological structure. Unlike the Complex-type ending in the submucosa which was always CGRP negative, 95% of all simple-type spinal endings in the submucosa were immunoreactive to CGRP (Fig. 10C & 10F). The characteristics of these simple-type endings in submucosa is shown in Table 1. To confirm all these three different types of endings did in fact ramify within the submucosa, we sharp dissected free the mucosa and submucosa from the full length colon. When the submucosa was removed, all three types of endings disappeared. The diameter of the axon at the nerve terminal of this class of spinal afferent was approximately 1 µm (Table 1).

### rIGLEs in submucosal ganglia

**Figure 11:**
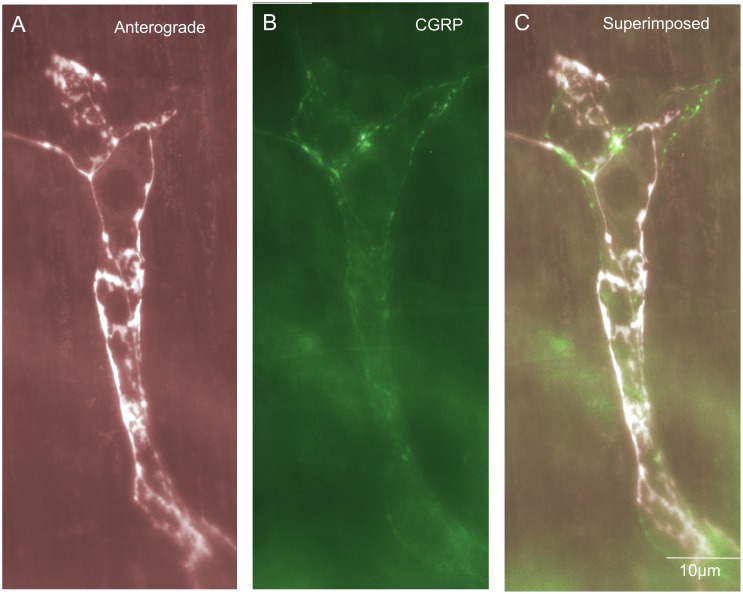
Anterograde labeling from lumbosacral DRG in vivo reveals on very rare occasions rectal intraganglionic laminar endings (rIGLEs) in submucosal ganglia. A, shows an anterogradely labeled image of an rIGLE in a submucosal ganglion. Panel B, CGRP immunoreactivity of the same region shown in panel A. The anterogradely labeled rIGLE is not CGRP immunoreactive. C, shows a superimposed image of A & B.

Rectal IGLEs were present but rarely identified in submucosal ganglia. They comprised a total of 1% of all endings in the large intestine and half of these were CGRP positive (Fig. 11E & F). rIGLEs have not been identified in submucosal ganglia to our knowledge. They did consist of flattened laminar endings that ramified extensively throughout ganglia (Fig. 11), as in myenteric ganglia [16]. The diameter of the axon at the nerve terminal of this class of spinal afferent was approximately 1 µm (Table 1).

### Mucosal spinal afferent endings

**Figure 12:**
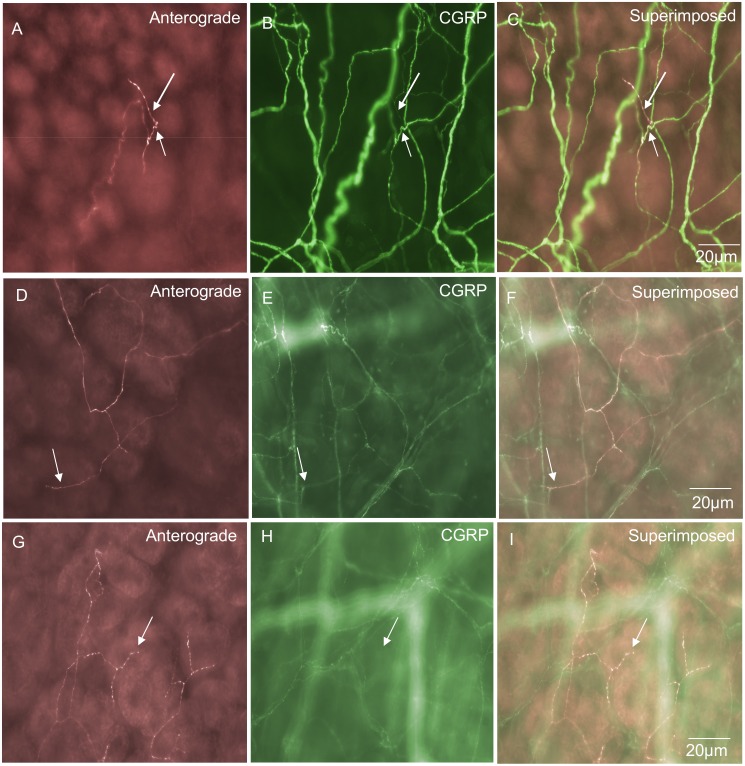
Anterograde labeling from lumbosacral DRG in vivo reveals spinal afferent nerve endings in the mucosa of three different mice. A, a single nerve axon ending is shown consisting of a bare unspecialized terminal with few varicosities. The arrows indicate the trajectory of this axon that is initially out of focus as it projects through the submucosa, then becomes in focus at the level of the mucosa. B, shows CGRP immunoreactivity of the region shown in panel A. The arrows indicate the same region of the axon that is in panel A. C, shows a superimposed image of panels B & A. The arrows indicate the anterogradely labeled axon is CGRP immunoreactive. D, shows a mucosal ending in a different animal consisting of a bifurcating axon, again with very few varicosities along its axon (see arrow). E, shows CGRP immunoreactivity of the region shown in panel D, see arrow. F, shows a superimposed image of panels E and D. This ending is CGRP positive, see arrow. G, shows another example from a different mouse of a varicose spinal afferent ending in the mucosa. The arrow shows a fine varicose ending. H, shows CGRP immunoreactivity of the region shown in G. The arrow indicates a lack of CGRP immunoreactivity of the ending shown by the arrow in panel G. I, shows a superimposed image of panels G and H. It can be seen that this mucosal ending is not CGRP positive (see arrows).

Nerve endings of spinal afferents were identified in the mucosa (Fig. 12). In total, from 45 mice studied, mucosal afferent endings occupied 11% of the total number of spinal afferent endings identified and 93% of all mucosal afferent endings were CGRP immunoreactivity (see Fig. 12B & E; Fig. 3B). A small proportion (7%) were not CGRP positive (Fig. 12H). Mucosal afferent endings consisted of either single axons that projected into the mucosa (Fig. 12A), or single varicose axons that branched into a number of axon terminal arbors in the mucosa (Fig. 12D & 12G). The characteristics of mucosally-projecting afferent endings is shown in Table 1. The diameter of the axon at the nerve terminal of this class of spinal afferent was approximately 1 µm (Table 1). To better visualize how spinal afferents innervated the mucosa, we used confocal microscopy to acquire a sequence of images throughout the full depth of the mucosa. Using these images, we developed a 3D movie reconstruction of a single mucosally-projecting axon, as it wraps around the base of the Crypts, then projects into the mucosa. This movie can be seen in Movie S1.

## Discussion

In mammals, spinal afferents are of supreme interest to us because it is clear that these are the sensory neurons that detect not only innocuous stimuli (such as those induced by mechanical and chemical stimulation) but also noxious (painful) stimuli and then transmit these sensory signals to the CNS. However, identifying all the different types of nerve endings of spinal afferents that innervate the viscera has been particularly challenging, largely because spinal afferents run alongside all the other classes of extrinsic efferent and afferent fibres. In this study, we have utilized a new surgical technique whereby it is now possible to visualize in high resolution (down to single varicosity level) all the different types of nerve endings of only spinal afferents that innervate different visceral organs. This study represents a technical breakthrough because until now, we have not been able to visualize *only* this class of sensory fibre in mammals, without also labeling all other classes of extrinsic nerves. As a result of this new technique, we present the first complete characterization of all the different types of spinal afferent nerve ending that innervate an internal organ of a mammal. Our findings have uncovered a remarkably complex range of distinct types of spinal afferent nerve endings that ramify within distinct layers of the large intestine of mice. Our findings suggest that compared to skin, visceral organs, such as the GI-tract appear to have evolved a considerably more extensive and diverse range of sensory endings.

A number of anterograde tracing studies have been made from nodose ganglia in mammals to identify the nerve endings of vagal afferents in visceral organs, such as the gastrointestinal tract [6], [7], [8], [9], [10], [26]. Consequently, the precise morphological features of vagal afferent endings has been extensively characterized [6], [7], [8], [9], [10]. Whilst there is some evidence that the vagus nerve can encode some nociceptive signals [27], it is generally considered that the vagus does not carry a large proportion of nociceptive signals to the CNS. In contrast, it is clear that spinal afferents play the major role in the detection of visceral and somatic sensation (including nociception), because lesions to the spinal nerves (or spinal cord) abolishes all sensation in mammals regardless of nociceptive or non-nociceptive origin [1], [2], [3]. Unlike the nodose and jugular ganglia which are readily accessible under surgery, DRG are much smaller and have been far more difficult to expose during a survival surgery on animals. We spent many months developing the survival surgery technique used in this study, whereby DRGs could be exposed in vivo in mice, injected with tracer, then recover successfully post-surgery. We studied many neuronal tracers using this technique and none were as successful at revealing the fine morphological features of nerve endings as the high molecular weight dextran biotin.

We found that if many DRGs were injected with dextran biotin and with large volumes of tracer, then many axons were labeled, which all typically overlapped each other within the large intestine. This made it almost impossible to follow the path of single axons and then directly correlate the trajectory of a single axon with its nerve terminal morphology. To get around this problem, we found that by injecting minute quantities of tracer (<300 nL dextran biotin) into small numbers of DRGs unilaterally (typical 1–2 DRGs) we were able to visualize very small numbers of axons in the large intestine. This offered a major advantage because it meant that we could directly visualize the point of entry of single spinal afferent axons in the large intestine then follow their path within the large bowel, to their ultimate sites of innervation. It was found that most axons entered through the serorsa and traversed through multiple myenteric ganglia before ending in discrete structures. In total, 13 distinct types of nerve ending were identified that ramified in distinct anatomical sites in the large intestine, many with unique morphologies that have not been described previously. Following injection of dextran biotin into DRG of numerous animals, we were able to develop a clear picture on which layers of the large intestine received the greatest proportion of spinal afferent nerve endings. The greatest innervation was in the myenteric ganglia, submucosa and circular muscle, which collectively comprised 82% of all the spinal afferent endings identified in the large bowel. The fewest number of spinal afferent nerve endings were found in the longitudinal muscle, submucosal ganglia and blood vessels.

In myenteric ganglia two major classes of spinal afferent endings were identified. The most common class was a novel type of ending that has not been described previously. This type of ending consisted usually of a single axon that entered a myenteric ganglion, then ramified throughout the ganglion, but lacked any complex morphological structure at its terminal ending (Fig. 1 & 2). Typically these types of nerve endings were varicose in nature and were described as intraganglionic varicose endings (IGVEs). In total, 66% of all IGVEs were CGRP positive. A fascinating common feature of many IGVEs was that the single parent axon that innervated a myenteric ganglion (and gave rise to IGVEs) often exited the ganglia for short distances, then returned back to the same ganglion before making extensive ramifications around many myenteric neurons (see Fig. 1D & 2F). In other animals, some IGVEs were weakly varicose and also terminated as a simple bare axon (e.g Fig. 2D). Rectal IGLEs were observed in myenteric ganglia of mouse large intestine, but they were far less common and only comprised 5% of the total proportion of nerve endings identified. rIGLEs have not been identified in the mouse intestine previously, but showed similar morphological features as those described in guinea-pig rectum [16]. That is, they consisted of flattened laminar endings that ramified throughout a single myenteric ganglion. None of the rIGLEs were immunoreactive to CGRP, which is consistent with rIGLEs in guinea-pig intestine [16]. Perhaps even more interesting was the observation, that, albeit very rarely, rIGLEs could be identified in submucosal ganglia (Fig. 11D).

### Mucosal endings

We are not aware of any previous studies that have identified spinal afferent nerve endings that innervate the intestinal mucosa of mammals. This information may be particularly useful for future studies where visceral hypersensitivity is induced in patients with inflammatory bowel disease, since the mucosa is a common site of inflammation. It has been presumed that spinal afferent endings innervate the mucosa since studies have shown that pressing the mucosa can evoke action potentials in spinal axons outside the gut wall. However, it has always been difficult to envisage that mechanically compressing the mucosa with Von Frey hairs could only activate nerve endings in the mucosa without also stimulating many other types of nerve endings in other anatomical structures such as the submucosa, muscle layers, or myenteric ganglia. The findings of the current study show that the spinal afferent nerve endings do project into the mucosa (see Movie S1) and occupy 11% of all the spinal afferent endings identified in the large intestine. They consist of single or branched fine varicose axons that ramify within the mucosa. The vast majority of mucosal endings (93%) were found to be CGRP positive.

### Different types of nerve endings in circular muscle

In a recent technical report, we identified a class of intramuscular spinal afferent ending that ramifies parallel to the circular muscle fibres, that we termed “branching-type” endings (see Fig. 1F in [24]. These endings arose from a single parent axon and branched to form multiple axons that ramified parallel to the CM fibres. In this study, we extended our understanding of this class of branching-type CM ending. We found that this class of nerve ending was again commonly identified. They represented 12% of all spinal afferent endings and 66% of these endings were immunoreactive to CGRP. This class of branching-type ending appears analogous to the intramuscular arrays (IMAs) that have been described in the upper GI-tract [6], [7] and intramuscular endings in the guinea-pig rectum [28], [29]. At present, the functional role of this class of intramuscular afferent is not clear in the mouse colon, but may represent the low threshold, wide dynamic range mechanoreceptors [12].

**Figure 13:**
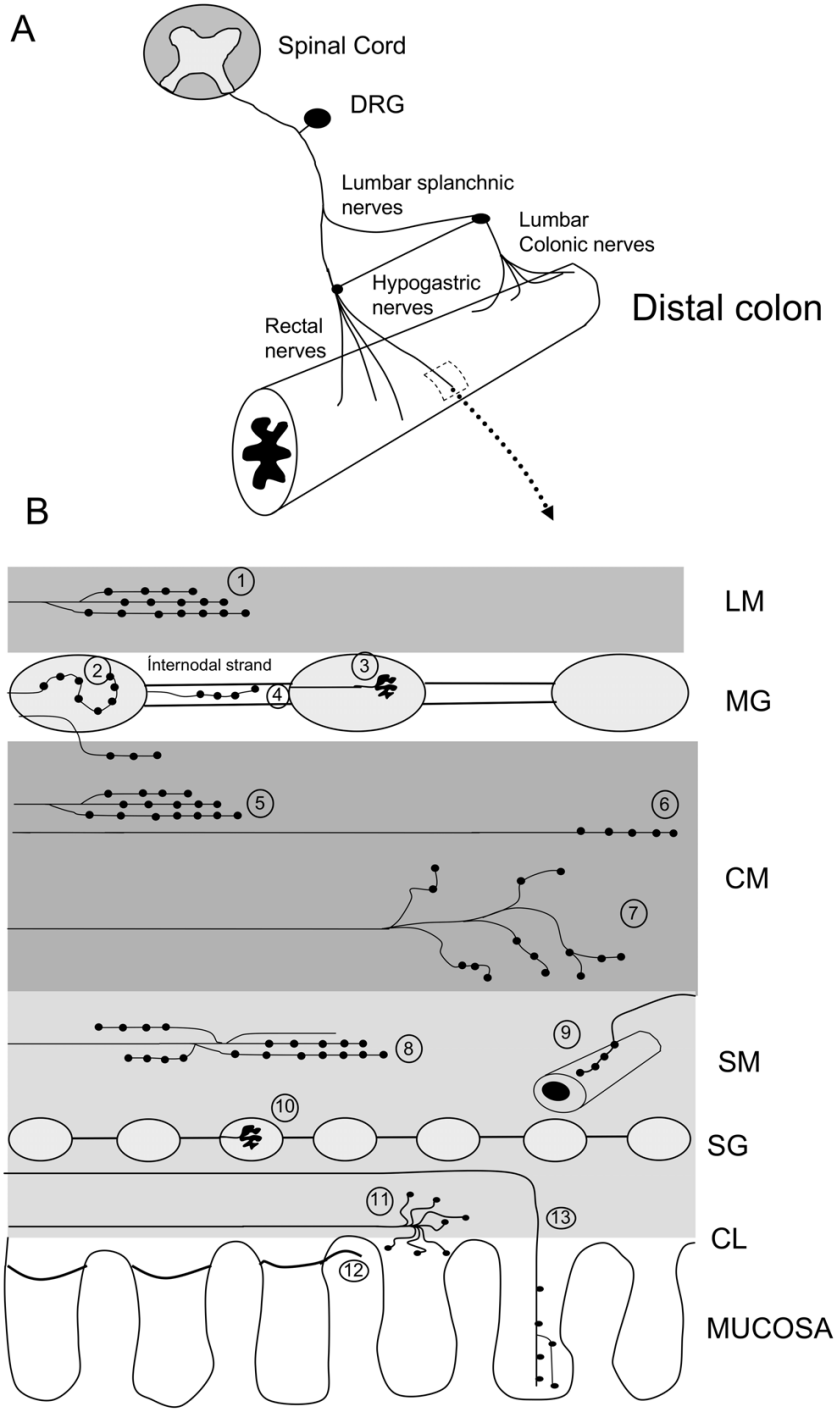
Diagrammatic representation of the different types of spinal afferent endings identified in the mouse large intestine, following minute injections of dextran biotin into lumbosacral DRGs in vivo. A, shows a schematic of the nerve pathways utilized to transport dextran biotin to the distal colon. B, shows that 13 distinct types of nerve endings were identified. 1 shows varicose endings in longitudinal muscle which occurred extremely rarely. Numbers 2 & 3, refer two types of endings identified in myenteric ganglia. 2 refers to intraganglionic varicose endings (IGVEs) which are common, while 3 refers to rectal intraganglionic laminar endings (rIGLEs) which are rare, 4 refers to varicose endings in internodal strands. 5 refers to “branching-type” endings in the CM layer that run parallel to the CM fibres, as in Figure 6D. 6 refers to “simple-type” afferent endings in the CM that consist of a single non-branching varicose axon, as in Figure 6A. 7 refers to the “complex-type” endings in the CM that arise from a single axon and ramify extensively throughout the CM layer in no preferential orientation, as in Figure 5A. 8, refers to “branching-type” spinal endings in the submucosa that ramify extensively in the rostral-caudal axis, as in figure 9A. 9, refers to endings on blood vessels. 10, refers to rIGLEs in submucosal ganglia. 11, refers to “complex-type” endings at the level of the Crypts of Lieberkhun, as in Figure 9E. 12, refers to simple type endings in the submucosa that consist of axon terminals that encircle the base of the Crypts with few or no varicosities, as in Figure 10A & 10D. 13, refers to nerve endings that innervate into the mucosa. KEY: LM refers to longitudinal muscle, MG = myenteric ganglion, CM = circular muscle, SM = submucosa, SG = submucosal ganglion and CL = Crypts of Lieberkuhn.

In this study, using our new anterograde tracing approach we have revealed a total of 13 distinct morphological classes of spinal afferent that innervate distinct anatomical layers of the large intestine (Fig. 13). Many of the types of nerve endings identified have not been identified previously. Some, or all, of the types of endings revealed here must contribute to the detection and transduction of noxious stimuli from the large intestine. It will be exciting to unravel which specific classes of these endings undergo modification during inflammation of the bowel wall and may underlie visceral hypersensitivity from the colon. Based on the complexity and diversity of the different types of spinal afferent endings identified in this study, it seems difficult to reconcile that only one class of ending underlies visceral hypersensitivity induced by colorectal inflammation.

Previous functional studies have proposed 4 functional classes of spinal afferent ending that run in the lumbosacral pathway to the large intestine and 5 classes in the thoracolumbar pathway [30]. This classification scheme was based on whether action potentials could be evoked in spinal afferent axons outside the large intestine, after stimuli were applied to compress full thickness segments of colon to activate sensory endings. The original classification scheme was: mucosal, muscular, muscular-mucosa and serosal for the pelvic/rectal pathway [30]. Whilst we could not identify any primary afferent nerve endings in the serosa of mice, consistent with previous anterograde tracing studies [29], we did find a remarkably complex array of different morphological types of spinal afferent ending in many other anatomical layers of the colorectum. We identified 1 major class of spinal afferent ending in the LM layer, 3 major classes in the CM layer, 2 major classes in myenteric ganglia, 1 class in internodal strands, 3 major classes in the submucosa (that includes 1 class in submucosal ganglia), 1 class innervating the Crypts of Lieburkuhn, 1 class in blood vessels, and 1 in the mucosa. Some of the morphological types of nerve ending identified must relate to the functional characteristics of spinal afferents that have been recorded electrophysiologically from the rectal and pelvic afferents.

## Conclusions

This study reveals a remarkable complexity and diversity of different morphological types of spinal afferent nerve endings that innervate distinct anatomical layers of the large bowel. Thirteen different types of primary afferent nerve endings were identified in the mouse large intestine (Fig. 13), whose cell bodies reside in DRG. Some, or all, of the nerve endings identified must underlie innocuous sensory neural reflexes that do not reach conscious levels, such as natural colorectal distension. Also, a population of identified nerve endings must also underlie the perception of pain from the large intestine, since lesions to the rectal/pelvic nerve pathway in vivo has been shown to abolish nociceptive reflexes induced by noxious stimuli [3]. A major challenge will be to ascertain which specific classes of primary afferent nerve endings underlies the detection of nociceptive and non-nociceptive stimuli in healthy and diseased bowel.

## Supporting Information

Movie S1 **This movie shows a 3D reconstruction of a single spinal afferent ending identified after anterograde labeling from L6 and S1 DRG in vivo.** The nerve ending ramifies around the Crypts of Leiburkhun and then projects into the mucosa. The grey colored area in the movie refers to the Crypts and the red is the single anterogradely labeled spinal afferent axon.
(MP4)
